# Supplementary material for: Urinary cell-free DNA is a versatile analyte for monitoring infections of the urinary tract
Source: Nat Commun. 2018 Jun 20;9:2412. doi: 10.1038/s41467-018-04745-0 (PMC6010457; doi:10.1038/s41467-018-04745-0)
Supplement: Supplementary file 1 — Supplementary Information [file 41467_2018_4745_MOESM1_ESM.pdf]

Supplementary Information for:

Urinary cell-free DNA is a versatile analyte for monitoring infections of the urinary tract

Burnham et al.

File includes:

Supplementary Figures 1-2

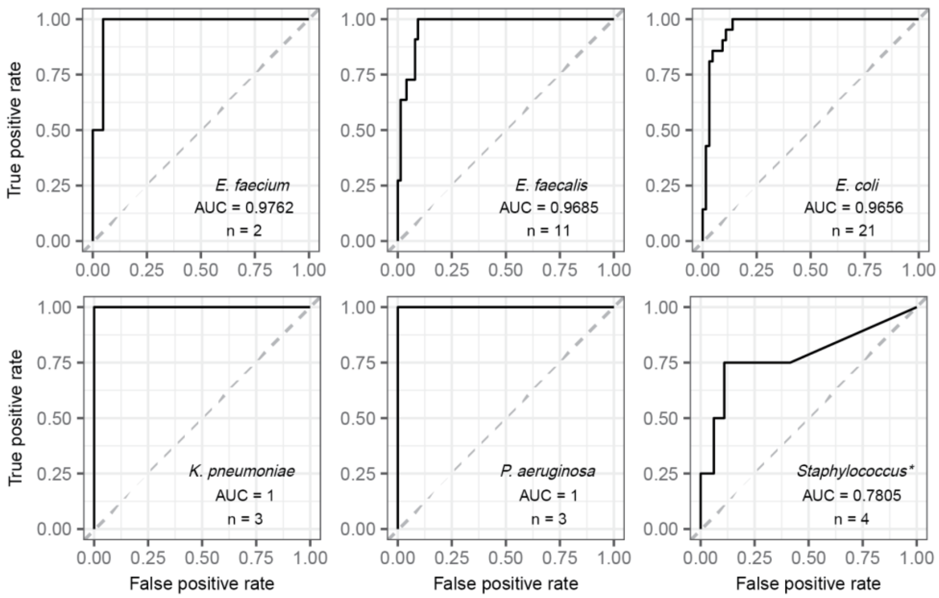

Supplementary Figure 1: **Analysis of performance of urinary cfDNA assay to identify bacterial UTI against conventional bacterial culture.** Receiver operating characteristic analyses of the performance of urinary cfDNA in identifying UTIs with the five suspected causative bacterial species and one suspected causative bacterial genus (marked with *). Area under curve = AUC, n = number of positive cultures. Total number of samples consisted of those clinically determined to have UTI based on positive urine culture, and those negative for UTI based on a bacterial count of < 10,000 CFU/mL in bacterial culture (total *n* = 86).

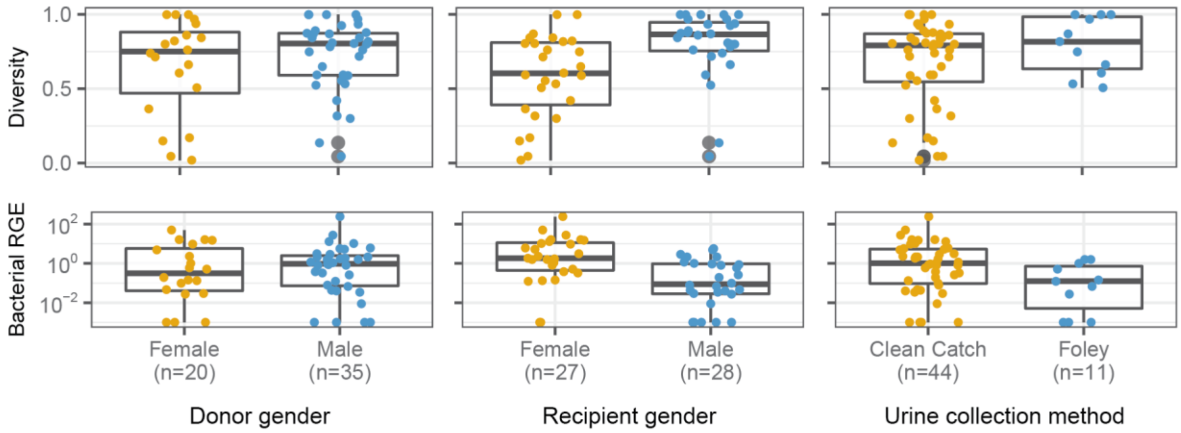

Supplementary Figure 2: Species-level diversity (Simpson index) and total relative genome abundance (RGE) as function of gender and urine collection method. The significance of clinical parameters on the bacteriome is tested using two-tailed Wilcox rank sum test for samples that were either UTI negative or taken within three days of transplantation (n = 55): donor gender ($p_{\text{diversity}}$ = 0.59 and p_{RGE} = 0.75), urine collection method ($p_{\text{diversity}}$ = 0.26 and p_{RGE} = 0.02), and recipient gender ($p_{\text{diversity}} = 1.7 \times 10^{-3}$ and $p_{\text{RGE}} = 3.2 \times 10^{-4}$).
