# Supplementary material for: Urinary cell-free DNA is a versatile analyte for monitoring infections of the urinary tract
Source: Nat Commun. 2018 Jun 20;9:2412. doi: 10.1038/s41467-018-04745-0 (PMC6010457; doi:10.1038/s41467-018-04745-0)
Supplement: Supplementary file 3 — Description of Additional Supplementary Files [file 41467_2018_4745_MOESM3_ESM.pdf]

Description of Additional Supplementary Files

File Name: Supplementary Data 1
Description: Clinical information including donor and recipient sex, number of days after transplant at which sample was collected, the results of bacterial culture, and the methods of urine capture are presented. Used to generate Figure 1B.

File Name: Supplementary Data 2
Description: BK viral nephropathy status and the relative genomic abundance (RGE) of BK polyomavirus cell-free DNA measured for each sample. Used to generate Figure 2A.

File Name: Supplementary Data 3
Description: The top 25 most abundant bacteria (by relative genomic abundance of cell-free DNA) for all samples with corresponding urine cultures. Used to generate Figure 2B.

File Name: Supplementary Data 4
Description: The relative genomic abundance of four uropathogenic bacteria (E. faecalis, E. coli, K. pneumoniae, and P. aeruginosa) for all samples with corresponding results from conventional urine culture. Used to generate Figure 2C.

File Name: Supplementary Data 5
Description: The relative genomic abundance of DNA viruses across all samples in the study. Used to generate Figure 2D.

File Name: Supplementary Data 6
Description: Samples used to generate microbial genome coverage plots for Figure 3A.

File Name: Supplementary Data 7
Description: Number of sequence alignments, Gini index, growth score, and goodness of fit across samples and bacterial species. Used to generate Figure 3B.

File Name: Supplementary Data 8
Description: DNA sequence alignments to genes conferring resistance to drugs of different antimicrobial classes. Used to generate Figure 4.

File Name: Supplementary Data 9
Description: Donor fraction estimates for samples from sex-mismatched donor-recipient pairs and corresponding clinical information. Used to generate Figure 5A and inset.

File Name: Supplementary Data 10
Description: Donor fraction estimates for samples from sex-mismatched donor-recipient pairs without clinically confirmed bacterial UTI. Used to generate Figure 5B.
